# Scientific skills in health services research – knowledge, utilization and needs for continuing education among staff at the University Hospital Tübingen

**DOI:** 10.3205/zma001692

**Published:** 2024-09-16

**Authors:** Hannah Richter, Anja Herrmann, Emily Piontkowski, Stefanie Joos, David Häske, Monika A. Rieger

**Affiliations:** 1 University Hospital Tübingen, Center for Public Health and Health Services Research, Tübingen, Germany; 2 University Hospital Tübingen, Institute for General Medicine and Interprofessional Care, Tübingen, Germany; 3 University Hospital Tübingen, Institute of Occupational Medicine, Social Medicine and Health Services Research, Tübingen, Germany

**Keywords:** research skills, research methods, university hospital, training courses, continuing education

## Abstract

**Objectives:**

As part of the further development of an existing training program on scientific skills for health services research at the University Hospital Tübingen, the aim of the study is to determine the level of knowledge, utilization and needs for continuing education among staff.

**Methods:**

In 2022, a semi standardized anonymous cross-sectional survey was conducted at the University Hospital Tübingen. The content of the questionnaire survey was the level of knowledge and utilization of various research methods, the need for continuing education on these and other healthcare research-related topics and the preferred training concept. The data analysis was carried out descriptively based on absolute and relative frequencies overall and grouped according to the scientific experience of the participants.

**Results:**

Participants’ self-assessment indicated that a proportion of them had research skills. However, the level of knowledge and utilization varied greatly with regard to different research methods. The 222 participants most frequently expressed a desire for continuing education in the preparation of meta-analyses (56%), questionnaire validation (43%) and -development (42%). There was also great interest in continuing education in the fields of project coordination (57%), third-party funded projects (46%) and science communication (45%).

**Conclusion:**

The survey highlights existing research skills and the need for methodological qualification in the field of healthcare research among the staff of the University Hospital Tübingen. The focus appears to be on evidence generation, methods of empirical social research and general research-related skills. The results will be used to design new training courses with a focus on health services research.

## Zusammenfassung

**Zielsetzung:**

Am Universitätsklinikum Tübingen (UKT) werden bestehende Fortbildungsangebote zu wissenschaftlichen Kompetenzen in der Versorgungsforschung weiterentwickelt. Ziel der Studie ist es den Kenntnisstand, Anwendung und Bedürfnisse hinsichtlich Fortbildungen bei den Beschäftigten zu ermitteln.

**Methodik:**

2022 wurde am UKT eine teilstandardisierte anonyme Querschnittsbefragung durchgeführt. Inhalt der Fragebogenerhebung war der Kenntnis- und Anwendungsstand hinsichtlich verschiedener Forschungsmethoden, das Bedürfnis nach Fortbildungen zu diesen und weiteren versorgungsforschungsnahen Themen sowie die präferierte Fortbildungskonzeption. Die Auswertung erfolgte deskriptiv anhand absoluter und relativer Häufigkeiten insgesamt sowie gruppiert nach wissenschaftlicher Erfahrung der Teilnehmenden.

**Ergebnisse:**

Die Selbsteinschätzung der Teilnehmenden zeigte, dass anteilig Forschungskompetenzen vorlagen. Der Kenntnis- und Anwendungsstand variierte stark in Bezug auf verschiedene Forschungsmethoden. Die 222 Teilnehmenden äußerten am häufigsten den Wunsch nach Fortbildungen zur Erstellung von Metaanalysen (56%), Fragebogenvalidierung (43%) und -entwicklung (42%). Großes Interesse bestand zudem an Fortbildungen zu Projektkoordination (57%), Drittmittelprojekte (46%) sowie Wissenschaftskommunikation (45%).

**Schlussfolgerung:**

Die Erhebung verdeutlicht bestehende Forschungskompetenzen und das Bedürfnis nach methodischer Qualifizierung im Bereich der Versorgungsforschung bei Beschäftigten des UKT. Hierbei scheint ein Schwerpunkt auf der Evidenzgenerierung, Methoden der empirischen Sozialforschung und allgemeinen forschungsbezogenen Kompetenzen zu liegen. Die Ergebnisse werden für die Konzeption neuer Fortbildungen mit Fokus auf Versorgungsforschung verwendet.

## 1. Introduction

Research at University Medicine traditionally focused on preclinical and clinical research. Actually, healthcare needs and challenges in the healthcare system are changing. These changes increasing the relevance of structural and system-oriented research areas such as public health, health systems research, epidemiology and healthcare research, so that in addition to translational research, the scientific focus on healthcare processes and population medicine is gaining in importance [1].

With regard to medical staff, there have been increasing calls in recent years for greater integration of scientific skills into the study of human medicine [2], [3], [4], [5], [6], [7]. The National Competence Based Catalogues of Learning Objectives for Undergraduate Medical Education (NKLM) 1.0 published in 2015 and the NKLM 2.0 adapted in 2021 ([https://nklm.de/zend/menu], retrieved on 24/11/2022) include a large number of skills and learning objectives in the field of medical-scientific skills that should be incorporated into the future design of the curricula of medical faculties. This area includes skills in researching and interpreting relevant literature and other sources of information as well as skills in the appropriate use of quantitative and qualitative methods of empirical social research, statistics and evidence-based medicine ([https://nklm.de/zend/menu], retrieved on 24/11/2022). Various degree programs in human medicine already describe the integration of scientific skills into their curricula, e.g. in the form of longitudinal science curricula or individual seminars and courses for students and doctoral candidates [8], [9], [10], [11], [12], [13], [14]. In addition to the described development in human medicine, the setting of university hospitals is characterized by increasing academisation and professionalisation in healthcare professions such as occupational therapy, midwifery, speech therapy, nursing and physiotherapy [15]. 

In 2019, the Center for Public Health and Health Services Research Tübingen was founded at the University Hospital Tübingen with the participation of the former Ministry of Social Affairs and Integration of the State of Baden-Württemberg with the aim of linking university medicine more closely with health services research and the public health service [16]. In addition to research projects in the field of health services research with a focus on and in cooperation with the public health service, the core tasks of the Center for Public Health and Health Service Research Tübingen include methodological consulting as well as the development and implementation of scientific-methodological qualification programs (method training). The method training is aimed at employees and junior researchers from university medicine and the public health service. Based on positive experiences with previous offerings [17], [18], workshops and regular research seminars have so far focused on three columns in particular: qualitative methods of empirical social research (including data collection using individual and focus group interviews, content analysis), quantitative methods of empirical social research (questionnaire construction and statistical analysis) and, among other things, the handling of routine data. 

A structured needs analysis has not yet been carried out. With the aim of further developing the Center for Public Health and Health Service Research Tübingen’s method training to suit the target group, needs were identified with regard to the content, methodological and didactic design of the method training with a view to the two target groups from university medicine and the public health service as part of the project “Infektionsschutz.Neu.Gestalten” (I.N.Ge) funded by the Federal Ministry of Health. This study deals with the following research questions in relation to employees at the University Hospital of Tübingen and in the context of health services research:

- What knowledge of scientific research methods exists?
- Which scientific research methods are used?
- What are the needs for continuing education in scientific research methods?

The results of a parallel survey of public health services will be presented separately [19]. 

## 2. Project description

### 2.1. Setting

The University Hospital Tübingen is one of five university hospitals in Baden-Württemberg and, with 1,585 beds, the third largest university hospital in Baden-Württemberg [20]. Research at the University Hospital Tübingen focuses on neurosciences, immunology and oncology, infection research, diabetes and vascular medicine ([https://www.medizin.uni-tuebingen.de/en-de/medizinische-fakultaet/forschung], accessed on 01/12/2022).

### 2.2. Data collection

From 12.07.-29.07.2022, a partially standardized anonymous cross-sectional survey was conducted online using the survey software UNIPARK (Tivan XI GmbH, Cologne, Germany). Recruitment took place by e-mail within the clinic and via notices in the staff canteens with information on voluntary participation. The survey was addressed to (future) scientific staff. Requirements for participation were an age of at least 18 years, sufficient understanding of the German language and affiliation with the University Hospital Tübingen or the Medical Faculty of the Eberhard Karls University of Tübingen. Based on the experience of previous years, in which the method training was attended by a large number of different disciplines, current or future scientific activity was not defined as an inclusion criterion. Instead, participation was made possible for all employees of the University Hospital Tübingen interested in scientific methods. The study has received a positive ethics vote from the responsible ethics committee of the University Hospital Tübingen and the Medical Faculty of the Eberhard Karls University of Tübingen (249/2022BO2).

### 2.3. Survey instrument

The interdisciplinary team developed a questionnaire with 22 items based on scientific literature (see attachment 1 ). Prior to the survey, a pre-test was carried out with four female academic staff from various institutions of the University Hospital Tübingen, whose feedback was incorporated into the finalization of the questionnaire. The final questionnaire comprised the survey of professional background, a question on self-assessment with regard to scientific research skills without limitation to a particular field of research (Likert scale) and questions on the level of knowledge and utilization with regard to quantitative and qualitative methods of empirical social research as well as with regard to other methods and study types of health services research (Likert-scaled: Answer options “I don’t know/have never heard of it”, “I know it, but haven’t used it yet”, “I have used it”). It also included questions on requests for continuing education in these and other science-related areas (e.g. submitting applications, scientific writing) and on software for data analysis. Finally, respondents were asked for their opinion on various organizational and didactic aspects of the training courses. In order to allow for answers that were not considered in the development of the questionnaire, the majority of the questions were formulated semi-openly with the option “other” and the possibility of free text input.

### 2.4. Data analysis

The data were analyzed using IBM SPSS Statistics 28 (IBM Inc., Armonk, NY, United States), whereby responses were partially aggregated (e.g. “low scientific research skills” and “basic knowledge of scientific research skills” to “moderate scientific research skills”). The results were presented descriptively using absolute and relative frequencies. All participants were included in the analysis, regardless of whether the questionnaire was answered completely. To make the article easier to read, individual formulations from the questionnaire are simplified (“scientific service and research”=“research”, “self-assessment of research competence”=“research competence”).

## 3. Results

The subsequent section will concentrate on the three most frequently mentioned aspects, for example the most frequently mentioned continuing education requests, for the overall sample and, categorized according to the participants’ scientific experience. A comprehensive overview of the results can be found in the attachment 2 , tables S1-S21).

### 3.1. Sample description

A total of 222 individuals participated in the survey, with 137 completing the survey. 100 participants (45%) indicated that they were engaged in research at the time of the survey. A total of 30 participants who were exclusively employed outside of research at the time of the survey indicated their intention to work in academia in the future. With regard to previous academic experience, the respondents indicated different durations of previous academic activities (see table 1 (Tab. 1)).

### 3.2. Self-assessment of research skills

A total of 182 (82%) stated that they had various levels of scientific research skills. Only 40 (18%) participants stated that they had no scientific research skills. At the time of the survey, they had never worked in science and the majority did not plan to work in science in the future. Those who were working in science at the time of the survey (n=97) rated their research skills as at least average. The longer participants had worked in academia in the past, the greater the proportion who reported having high academic research skills (see table 2 (Tab. 2)).

### 3.3. Knowledge of scientific research methods

The implementation of real laboratories (67%), Patient Reported Outcomes Experience Measures (64%) and discourse analyses (60%) were the research methods with which the fewest participants were familiar. In contrast to those with less or no experience, those who had already been working in research for over 10 years indicated a lack of knowledge of qualitative evaluation methods such as the documentary method and biographical research methods (see table 3 (Tab. 3)). 

### 3.4. Utilization of scientific research methods

The collection of primary data (66%), questionnaire development (55%) and hypothesis testing (52%) were the research methods that most participants had already used. With increasing scientific experience, a larger proportion stated that they had already used these methods. While participants who were not or had not previously worked in science indicated in particular the use of survey methods, quantitative evaluation methods such as hypothesis testing, correlation and regression analyses were among the three most frequently used methods for those with scientific experience (see attachment 2 , tables S1-S7).

### 3.5. Requirements for continuing education in scientific research methods

Interest in continuing education courses was particularly expressed in meta-analyses (56%), questionnaire validation (43%) and development (42%) as well as model quality control (42%) and creating reviews (narrative, scoping, etc.) (42%). While nonscientists were most interested in training in data collection methods, those who were not yet scientists but intended to be in the future were most interested in training in evaluation methods. Those already working in research were particularly positive about training in meta-analysis and more complex quantitative evaluation methods, such as hierarchical models and structuring methods (see table 4 (Tab. 4)).

### 3.6. Requirements for continuing education on research-related topics

continuing education in research-related topics was particularly requested for project coordination (57%), third-party funding application/administration and overview (46%) and science communication (45%). Above all, the desire for continuing education in project coordination was expressed regardless of scientific experience. Those with little or no scientific experience were also interested in continuing education on basics such as developing research questions and scientific writing. Those with scientific experience, on the other hand, expressed a particular desire for continuing education on data protection issues in research, scientific communication and specific applications (see table 5 (Tab. 5)). 

### 3.7. Requirements for continuing education on data processing software

The majority of respondents were interested in continuing education in data processing software with regard to SPSS (63%), SAS (42%) as well as STATA (37%) and MAXQDA (37%). Even though a proportion of up to 54% expressed interest in continuing education on MAXQDA, particularly among those with less scientific experience, there was a greater interest in continuing education on statistical than on qualitative data analysis programs, regardless of scientific experience (see attachment 2 , tables S14-S20).

### 3.8. Continuing education concept 

When asked how methodical training should be organized regardless of the COVID-19 pandemic, most respondents indicated a desire for blended learning programs (42%). 85% also wanted a certificate of attendance. With a few exceptions, these preferences were independent of scientific experience (see attachment 2 , table S21). 

## 4. Discussion

In Germany, health services research has so far mainly been taught in human medicine and health science programs [21]. However, surveys among students have shown that medical students in particular rate their scientific skills as rather low [22]. In another survey, below-average numbers of human medicine students stated that their previous studies had supported them in terms of scientific methodological skills and the ability to carry out independent research [23]. Even though there is an increasing integration of scientific skills into the curricula of medical programs in Germany [8], [9], [10], [11], [12] and an increasing professionalization and academization of healthcare professions [15], it can be assumed that there is currently a need for continuing education in scientific skills in the setting of university hospitals.

The results of this study enable the further development of method training at the Center for Public Health and Health Service Research Tübingen. The results of the self-assessment of research skills indicate that, from the respondents’ perspective, research skills are already proportionately present, particularly among those already engaged in scientific research. However, the majority of junior researchers did not rate their research skills as high. As expected, nonscientists in particular indicated low research skills. The fact that, despite a heterogeneous sample, at least a quarter of the participants expressed a desire for continuing education in each of the research methods included in the survey illustrates the need for methodological training in the field of health services research at the University Hospital Tübingen. The participation of non-scientific employees in the survey also indicates that there is also interest in topics and methods of healthcare research outside of primarily scientific activities at the University Hospital Tübingen. The evaluation according to scientific experience also shows that different groups of people interested in method training have different needs, which should be considered in the design, particularly with regard to expected prior knowledge.

A conspicuous focus on quantitative methods in the most frequently requested continuing education courses can be explained on the one hand by the clinical and basic research focus of the University Hospital Tübingen ([https://www.medizin.uni-tuebingen.de/en-de/medizinische-fakultaet/forschung], accessed on 01/12/2022). With regard to the differences in the level of knowledge and utilization between quantitative and qualitative research methods, the focus can also presumably be attributed to the long dominance of quantitative methods in health services research [24] and in health services research studies [21]. However, when designing the method training, it is important to consider that qualitative research approaches are becoming increasingly important due to the changing spectrum of diseases towards chronic diseases and research into topics such as quality of life and well-being [25]. In order to do justice to the complementary nature of quantitative and qualitative research strategies [25], it is also planned to teach research skills in both areas in future method training at the University Hospital Tübingen. The methods training was expanded to include new workshops, e.g. on the topics of meta-analyses, literature research and evaluation of literature as well as complex interventions. Existing events on desired topics such as questionnaire development and interviews were retained. The inclusion of a workshop on questionnaire validation is planned and is currently being organized. The demand for continuing education on research-related topics has been met with a new format, short method impulses (see table 6 (Tab. 6)). 

With regard to the design of the continuing education courses, most participants preferred blended learning programs, although online and in-person events also received approval. Considering the different potentials of the different types of events, such as greater efficiency [26] and more intensive peer-to-peer exchange [26], [27] for in-person events as well as flexibility in terms of location and time [26], [27] and reduced travel, costs and time expenditure for online offerings [27], it will be necessary to weigh up which potentials should be used when designing training courses in the future. In addition, e-didactic methods can be used to reduce the limitations of online formats [28]. In our case, another target group of the method training, employees from the public health service, must also be considered in the planning, which can benefit in particular from the spatial flexibility of online units. A blended learning format has already been tested as part of the method training and has been well received. Further offers are being planned.

In order to assess whether the modified version of the Center for Public Health and Health Service Research Tübingen methods training meets the needs and requirements of the target groups and to ensure continuous further development, an evaluation of the events was established from fall 2023. As part of the evaluation, feedback will be obtained on the event attended (e.g. quantity of content taught, quality of content taught and didactics) and on the method training as a whole (e.g. relevance, variety of topics and organization).

### Limitations

When interpreting the present results, it should be noted that the subjective assessments of the participants were depicted and the term “scientific research skills” was not defined in advance. It should also be noted that only 137 participants completed the survey. As we have hardly any information on all employees at the University Hospital Tübingen and no socio-demographic data was collected in the survey for data protection reasons, it is not possible to assess whether the participants are representative of the employees at the University Hospital Tübingen. Furthermore, it cannot be ruled out that people interested in continuing education on scientific methods in particular took part in the survey. It should also be noted that the survey was conducted exclusively at the University Hospital Tübingen. The heterogeneity of research focuses and the structure of medical training should be considered when transferring the results to other university hospitals or settings.

## 5. Conclusion

The present survey points to the need for continuing education in scientific skills for health services research in the setting of the University Hospital Tübingen. The detailed recording of the level of knowledge and utilization as well as the wishes regarding the content and design of continuing education courses for teaching scientific skills have already been incorporated into the further development of the method training. The integration of further courses on identified topics is planned. The continuous further development will also be supplemented by the establishment of an evaluation of the participants.

## Notes

### Authorship

The authors David Häske and Monika A. Rieger share the last authorship.

### Authors’ ORCIDs

- Hannah Richter: [0000-0002-9672-2184]
- Anja Herrmann: [0009-0009-5438-8438]
- Emily Piontkowski: [0009-0000-5372-844X]
- Stefanie Joos: [0000-0002-5810-529X]
- David Häske: [0000-0001-5190-3937]
- Monika A. Rieger: [0000-0002-7855-3663]

### Funding

This work was supported as part of the project “Infektionsschutz.Neu.Gestalten” (I.N.Ge) by the Federal Ministry of Health under the funding code ZMI1-2521FSB111. The Institute of Occupational Medicine, Social Medicine and Health Services Research, University Hospital Tübingen, receives institutional funding from the Verband der Metall- und Elektroindustrie Baden-Württemberg e.V. (Südwestmetall). 

## Acknowledgements

The Institute for Clinical Epidemiology and Applied Biometry at the University of Tübingen provided methodological advice for this study. We would like to thank Dr. You-Shan Feng for her support. We would also like to thank Dr. Achim Siegel from the Institute of Occupational Medicine, Social Medicine and Health Services Research for his support in developing the questionnaire and Miriam Colombo, Carina Klocke and Anna-Jasmin Wetzel from the Institute of General Practice & Interprofessional Care for their support in developing the questionnaire and for their feedback on the results. We would like to thank Ms. Nastaran Jafari for her support with the English translation.

## Competing interests

The authors declare that they have no competing interests. 

## Supplementary Material

Questionnaire

Additional tables

## Figures and Tables

**Table 1:**
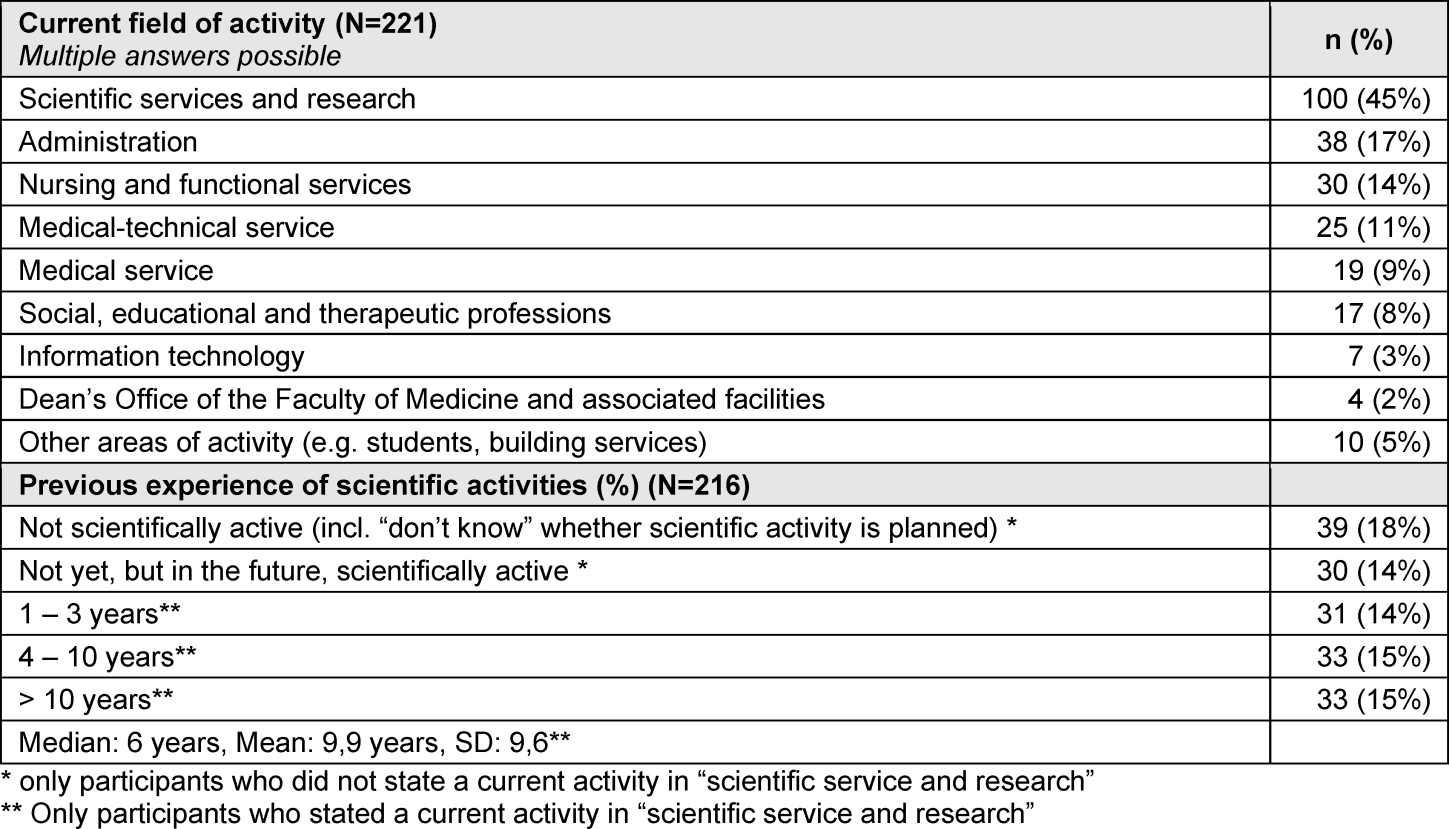
Sample description

**Table 2:**
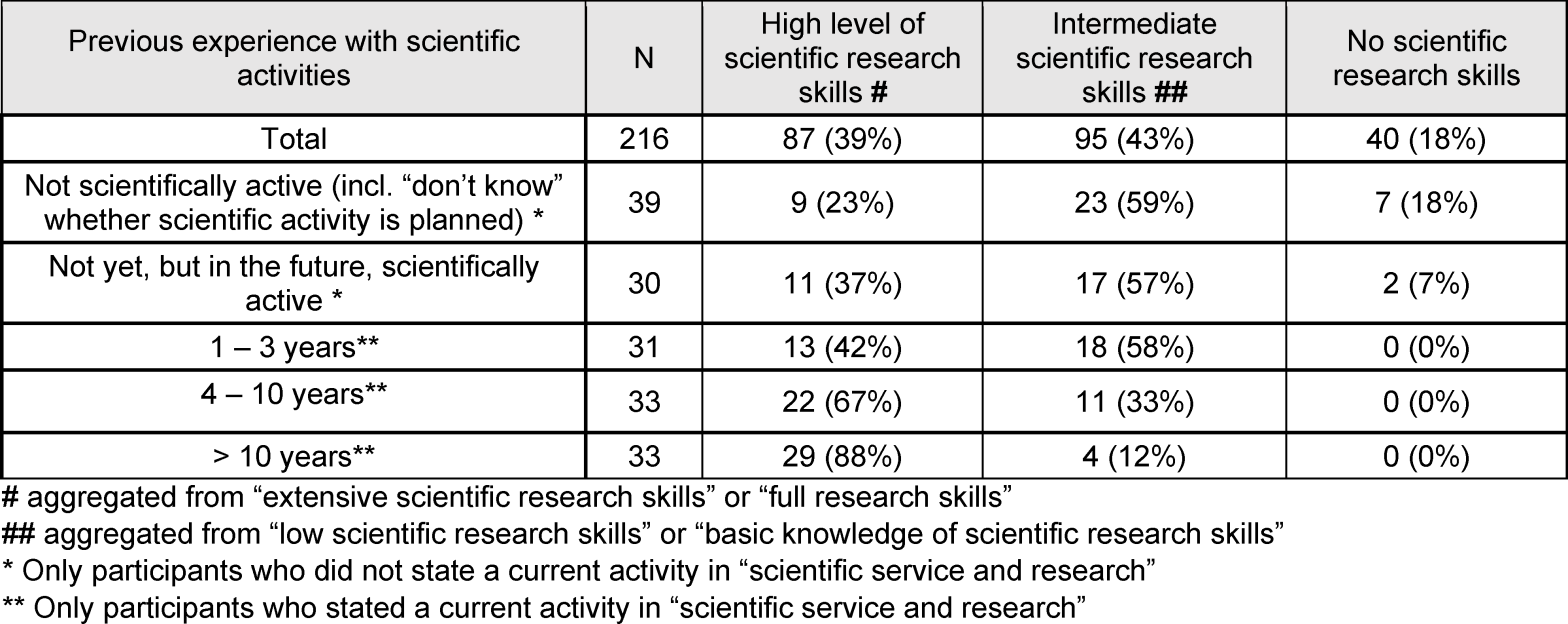
Self-assessment of scientific research skills according to previous experience with scientific activities

**Table 3:**
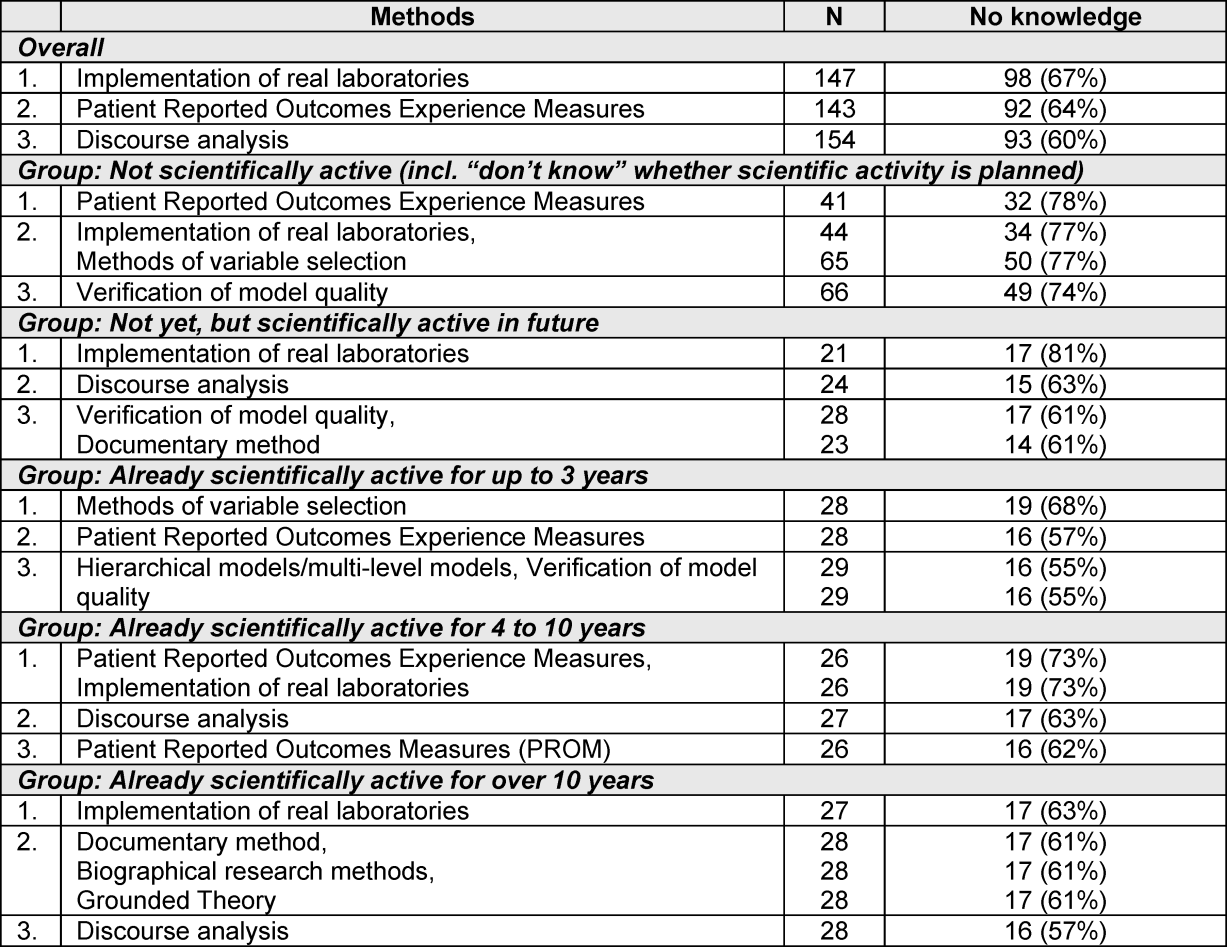
The three most frequently mentioned lack of knowledge in scientific research methods overall and grouped by participants’ scientific experience

**Table 4:**
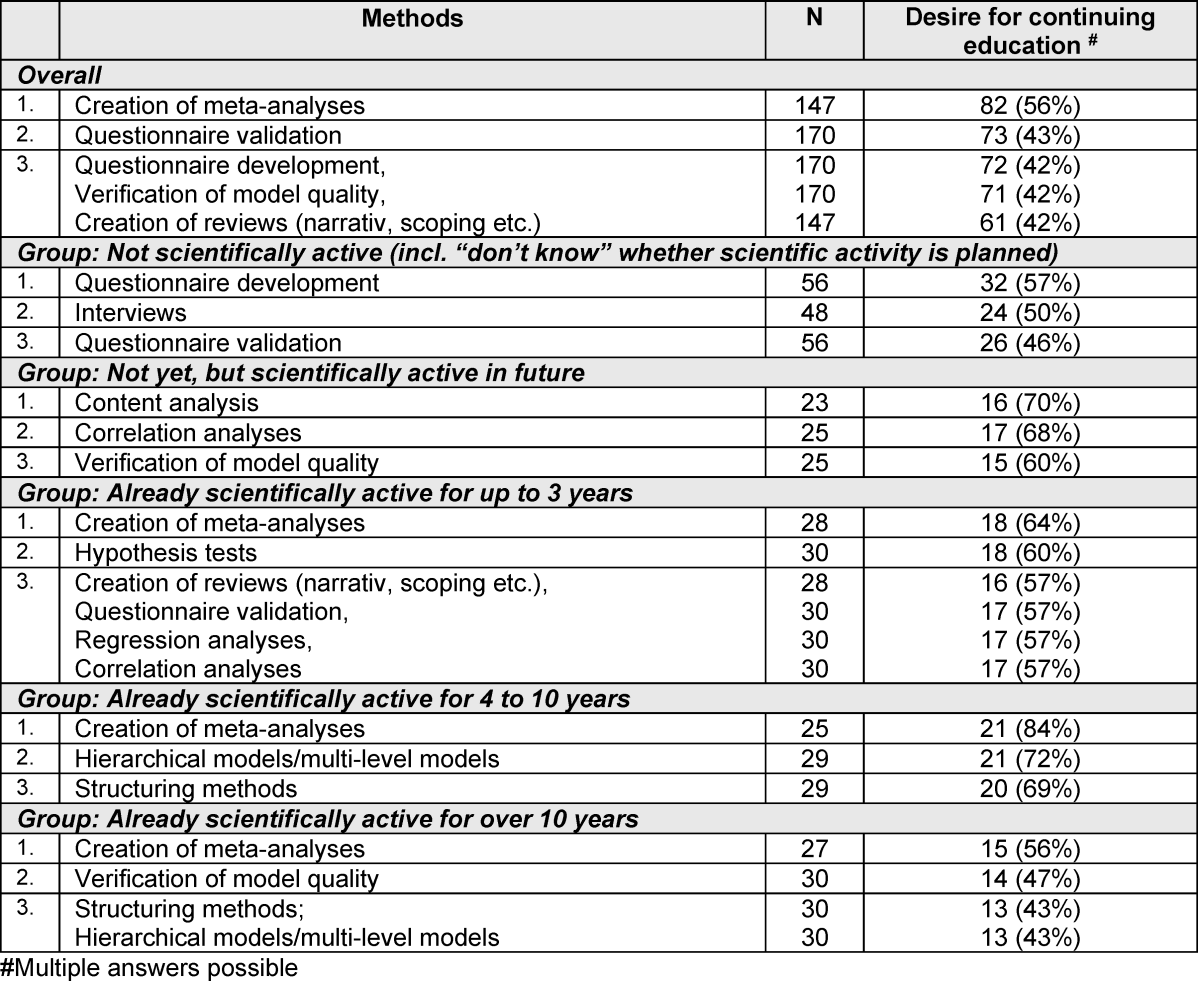
The three most frequently mentioned continuing education requests overall and grouped by participants’ scientific experience

**Table 5:**
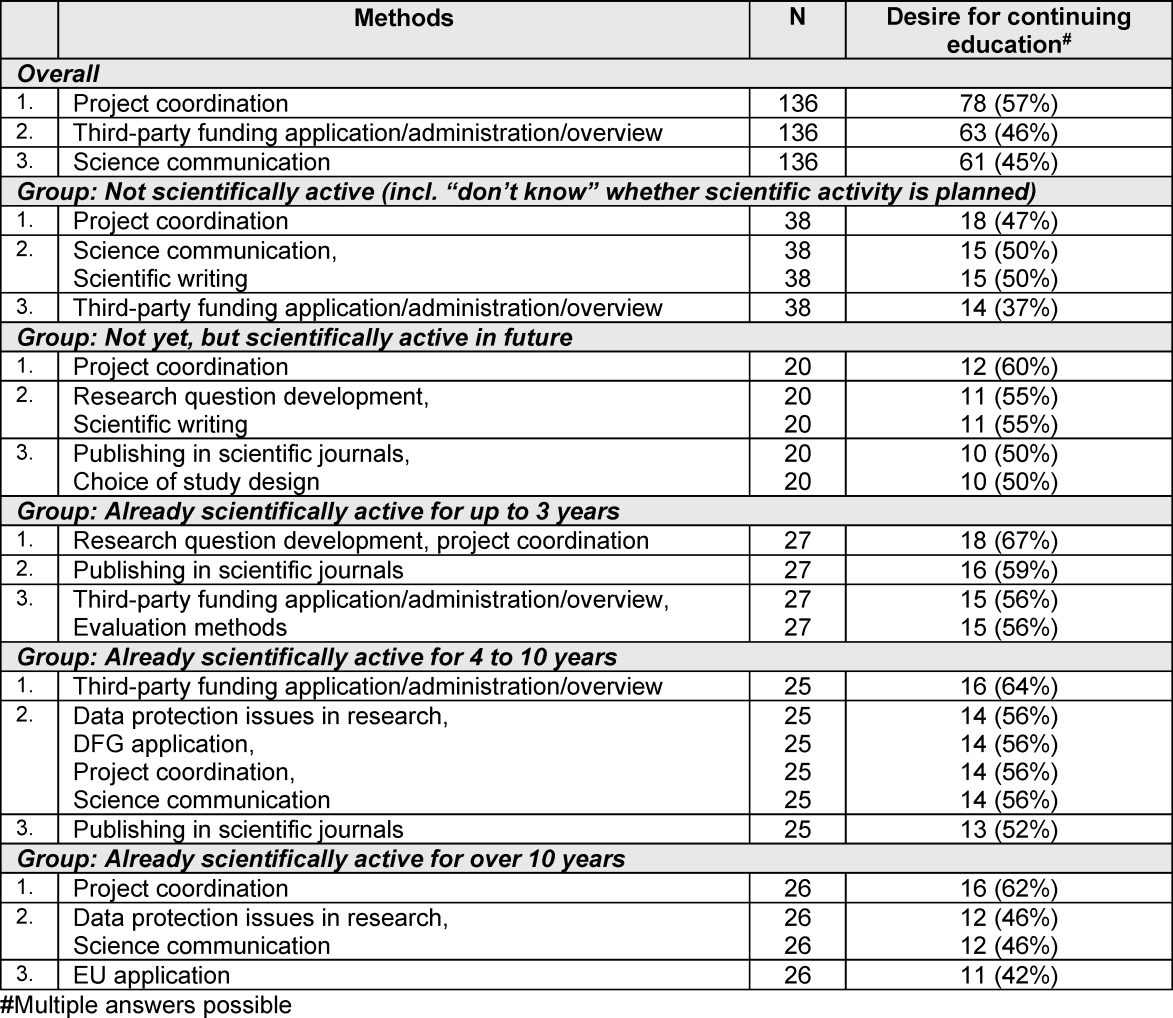
The three most frequently mentioned continuing education requests with regard to research-related topics overall and grouped by participants’ scientific experience

**Table 6:**
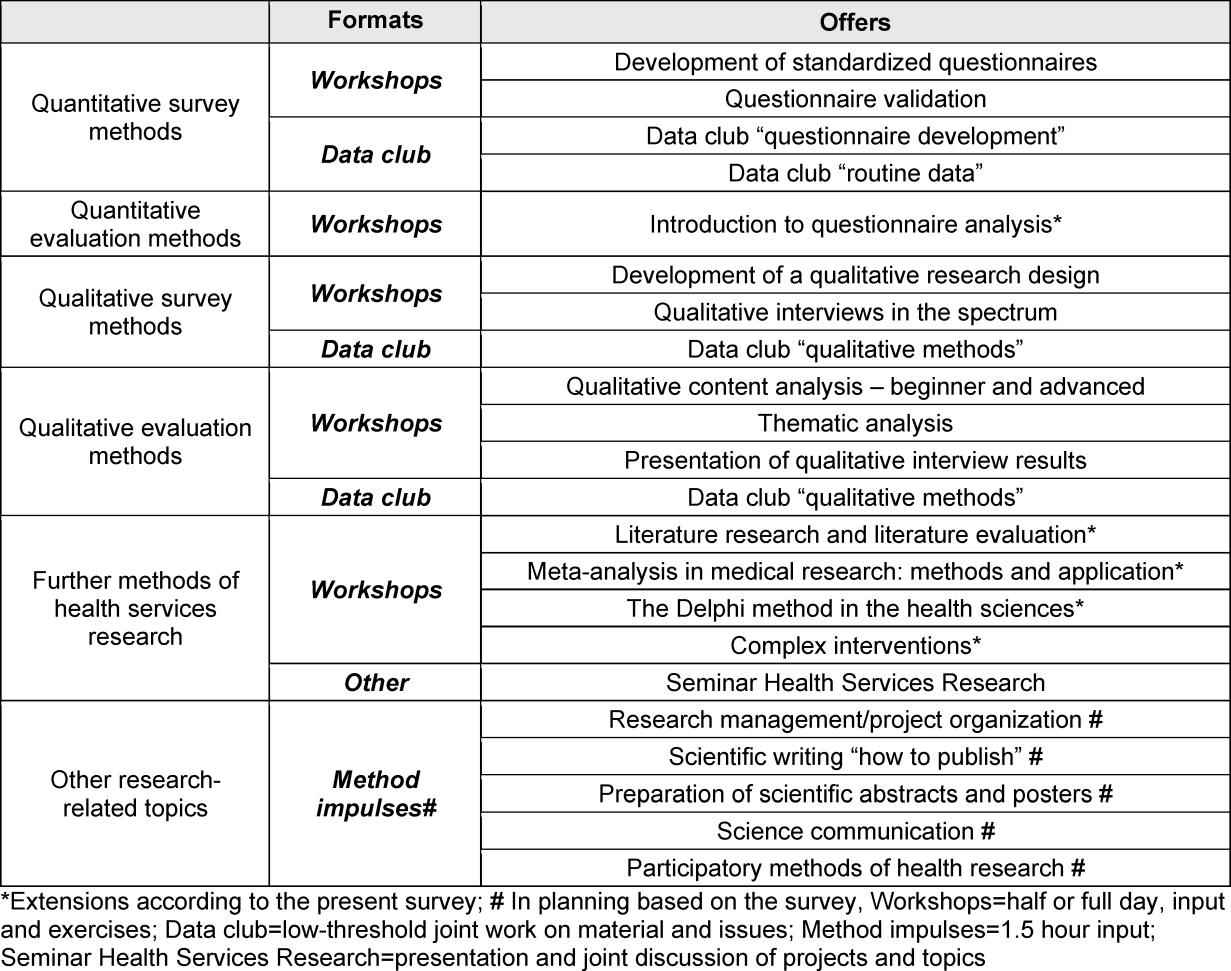
Method training offers of Center for Public Health and Health Service Research Tübingen, as of: December 2023
