# Supplementary material for: Scientific skills in health services research – knowledge, utilization and needs for continuing education among staff at the University Hospital Tübingen
Source: GMS J Med Educ. 2024 Sep 16;41(4):Doc37. doi: 10.3205/zma001692 (PMC11474650; doi:10.3205/zma001692)
Supplement: Questionnaire [file JME-41-37-s-001.pdf]

Attachment 1: Questionnaire

1. Basic questions / Background

In the following questions we would like to find out about your possible scientific background

How would you rate your scientific research skills?
(Indicate your personal, subjective assessment)

- o I do not have any scientific research skills
- o I have limited scientific research skills
- o I have a basic knowledge of scientific research skills
- o I have extensive scientific research skills
- o I have comprehensive scientific research skills

In which area do you currently work? (multiple answers possible)

- o Nursing and functional services
- o Medical service
- o Medical-technical service
- o Social, educational & therapeutic professions
- o Administration
- o Information technology
- o Scientific service and research
- o Dean's Office of the Faculty of Medicine and associated facilities
- o Other, namely: ______________________

Filter question for answer “Scientific service and research”

How many years have you been or are you currently working in academia?
Please enter whole years (e.g. 1,5,12, etc.) If you have not yet been scientifically active for a full year, please enter the number 1

____

Filter question for answers other than “Scientific service and research”

Do you plan to pursue a career in science?

- o Yes
- o No
- o I don't know

2. Knowledge and utilization of research methods and desire for continuing education

A wide range of methods are used in health services research. Quantitative methods for numerical visualisation have a firm place here.

How familiar are you with the following **quantitative** survey methods?

	I don't know it, I've never heard of it	I know it, but I haven't used it yet	I have already used it
Questionnaire development (e.g. standardised survey, multiple choice, closed)			
Questionnaire validation			
Collection of primary data (data collected for research purposes)			
Handling of secondary data (e.g. handling of data that was not primarily collected for research purposes but is then used)			
Other, namely:			

How familiar are you with the following **quantitative** evaluation methods / test procedures?

	I don't know it, I've never heard of it	I know it, but I haven't used it yet	I have already used it
Hypothesis tests (e.g. t-test, chi-square test, Mann-Whitney U-test, Wilcoxon test)			
Correlation analyses (e.g. Pearson correlation analysis, Spearman rank correlation)			
Regression analyses (e.g. simple linear regression, multiple regression, multinomial regression, ordinal regression, Cox regression, Poisson regression)			
Methods of variable selection (e.g. forward selection, backwards selection, LASSO)			
Structuring methods (e.g. factor analysis, principal component analysis, cluster analysis)			
Verification of model quality (e.g. bootstrap, cross-validation, AIC, BIC)			
Hierarchical models/multi-level models			
Other, namely:_______________			

Where would you like to see training formats in **quantitative** research?
Multiple answers are possible.

- o Questionnaire development (e.g. standardised survey, multiple choice, closed)
- o Questionnaire validation
- o Collection of primary data (data collected for research purposes)
- o Handling secondary data (e.g. handling data that was not primarily collected for research purposes but is then used)
- o Hypothesis tests (e.g. t-test, chi-square test, Mann-Whitney U-test, Wilcoxon test)
- o Correlation analyses (e.g. Pearson correlation analysis, Spearman rank correlation)
- o Regression analyses (e.g. simple linear regression, multiple regression, multinomial regression, ordinal regression, Cox regression, Poisson regression)
- o Methods of variable selection (e.g. forward selection, backwards selection, LASSO)
- o Structuring methods (e.g. factor analysis, principal component analysis, cluster analysis)
- o Verification of model quality (e.g. bootstrap, cross-validation, AIC, BIC)
- o Hierarchical models/multi-level models
- o Other, namely: _______________
- o None

Qualitative methods also play a central role in healthcare research. These are not numerical, but are recorded verbally, visually or textually, for example.

How well do you know the following **qualitative** <u>survey</u> methods?

	I don't know it, I've never heard of it	I know it, but I haven't used it yet	I have already used it
Interviews (e.g. guided or problem-centred interviews etc.)			
Group discussion methods e.g. focus group interviews etc.)			
Participatory survey methods, e.g. with the active involvement of study participants in planning or design			
Participatory observation			
Document analysis			
Other, namely: ______________			

How well do you know the following **qualitative** <u>evaluation</u> methods?

	I don't know it, I've never heard of it	I know it, but I haven't used it yet	I have already used it
Content analysis			
Grounded Theory			
Thematic analysis			
Documentary method			
Biographical research methods			
Discourse analysis			
Other, namely:			

In which area would you prefer a training format in **qualitative** research?
Multiple answers are possible

- Interviews (e.g. guided or problem-centred interviews etc.)
- Group discussion methods e.g. focus group interviews etc.)
- Participatory survey methods, e.g. with the active involvement of study participants in planning or design
- Participatory observation
- Document analysis
- Content analysis
- Grounded Theory
- Thematic analysis
- Documentary method
- Biographical research methods
- Discourse analysis
- Other, namely: _______________
- None

In health services research and evidence-based medicine, the following methods are frequently used to answer scientific questions.
Please indicate how familiar you are with the following examples.

	I don't know it, I've never heard of it	I know it, but I haven't used it yet	I have already used it
Complex interventions			
Patient Reported Outcomes Measures (PROM)			
Patient Reported Outcomes Experience Measures (PREM)			
Creation of reviews (narrative, scoping etc.)			
Preparation of systematic reviews			
Creation of meta-analyses			
Carrying out real laboratories			
Other, namely:			

In which of the methods mentioned would you be interested in further training?
Multiple answers possible

- Complex interventions
- Patient Reported Outcomes Measures (PROM)
- Patient Reported Outcomes Experience Measures (PREM)
- Creation of reviews (narrative, scoping etc.)
- Preparation of systematic reviews
- Creation of meta-analyses
- Carrying out real laboratories
- Other, namely: _______________
- None

How do you usually analyse collected data (qualitatively or quantitatively)?
Multiple answers are possible

- I do not analyse any data
- I analyse the data myself using appropriate methods
- I give it to a specific specialist department, e.g. Institute for Biometry at the University Hospital or the Faculty of Medicine
- I give it to an external provider
- Other, namely: _______________

For which data processing software do you see a need for further training?
If you do not know a software and do not plan to use it in the near future, please tick 'I have no need'.

	I have no need	I would like to learn the basics	I would like an advanced course	I would like a basic course + advanced course in combination
MAXQDA				
Atlas.ti				
F4				
SPSS				
SAS				
STATA				
Other, namely:				

Further wishes and requirements

Which key topics would you like to see covered in a training format?
Multiple answers possible.

- Project coordination / project management
- Third-party funding application / administration / overview
- DFG application
- BMBF application
- EU application
- Choice of study design
- Development of research questions
- Evaluation methods
- Evaluation of complex interventions
- Process evaluation models and practical procedures
- Scientific writing
- Publishing in scientific journals
- Poster creation
- Research ethics and legal issues
- Health economics
- Topics relating to health services research (settings, methods, relevance)
- Epidemiology
- Data protection issues in research
- Science communication
- Other, namely: _______________
- None

Framework conditions and organisation of the continuing education formats
How many times a year do you usually attend continuing education courses on research methods?<br>Please enter only whole numbers. e.g. approx. [X] times per year<br>_______________
Where do you look for training opportunities?<br>Multiple answers are possible.<br>o At the University Hospital Tübingen<br>o At the Eberhard Karls University of Tübingen<br>o Other universities and colleges<br>o Subject-specific networks / mailing lists<br>o I am not looking for it<br>o Other, namely: _______________
How should methodological training be organized?<br>Regardless of the corona pandemic.<br>o Face-to-face<br>o Online<br>o Blended learning programs (e.g. combination of face-to-face units, online lectures and online recordings with practical exercises that are completed through self-study)<br>o No preference
Would you like to receive a certificate of attendance?<br>o Yes<br>o No<br>o No preference
Do you generally collect continuing education points?<br>o Yes<br>o No
Filter question if answer is “Yes”<br>If so, would you still attend the continuing education course even if there were no further continuing education points available for it?<br>o Yes<br>o No
Do you know the Center for Public Health and Health Services Research (ZÖGV) at the University Hospital of Tübingen?<br>o Yes<br>o No
