# Supplementary material for: Scientific skills in health services research – knowledge, utilization and needs for continuing education among staff at the University Hospital Tübingen
Source: GMS J Med Educ. 2024 Sep 16;41(4):Doc37. doi: 10.3205/zma001692 (PMC11474650; doi:10.3205/zma001692)
Supplement: Additional tables [file JME-41-37-s-002.pdf]

Attachment 2: Additional tables

Table S1: Proportion of participants with a desire for continuing education in scientific research methods and overall level of knowledge and utilization

	Methods	N	Desire for continuing education #	N	No knowledge	Knowledge, but no utilization yet	Already used
Quantitative survey methods	Questionnaire validation	170	73 (43%)	197	17 (9%)	124 (63%)	56 (28%)
	Questionnaire development	170	72 (42%)	198	8 (4%)	81 (41%)	109 (55%)
	Dealing with secondary data	170	57 (34%)	198	18 (9%)	82 (41%)	98 (50%)
	Collection of primary data	170	54 (32%)	197	14 (7%)	54 (27%)	129 (66%)
Quantitative evaluation methods	Verification of model quality	170	71 (42%)	186	101 (54%)	65 (35%)	20 (11%)
	Structuring methods	170	67 (39%)	186	61 (33%)	79 (43%)	46 (25%)
	Hierarchical models/multi-level models	170	66 (39%)	185	91 (49%)	71 (38%)	23 (12%)
	Methods of variable selection	170	65 (38%)	183	107 (59%)	60 (33%)	16 (9%)
	Regression analyses	170	62 (37%)	186	49 (26%)	62 (33%)	75 (40%)
	Correlation analyses	170	62 (37%)	186	56 (30%)	52 (28%)	78 (42%)
	Hypothesis tests	170	59 (35%)	184	51 (28%)	38 (21%)	95 (52%)
Qualitative survey methods	Interviews	154	60 (39%)	162	13 (8%)	85 (53%)	64(40%)
	Group discussion method	154	59 (38%)	159	30 (19%)	94 (59%)	35 (22%)
	Document analysis	154	50 (33%)	162	31 (19%)	74 (46%)	57 (35%)
	Participatory observation	154	50 (33%)	162	42 (26%)	88 (54%)	32 (20%)
	Participatory survey methods	154	42 (27%)	162	51 (32%)	83 (51%)	28 (17%)
Qualitative evaluation methods	Content analysis	154	59 (38%)	149	45 (30%)	52 (35%)	52 (35%)
	Thematic analysis	154	49 (32%)	154	69 (45%)	54 (35%)	31 (20%)
	Documentary method	154	46 (30%)	154	75 (49%)	59 (38%)	20 (13%)
	Biographical research methods	154	40 (26%)	155	82 (53%)	67 (43%)	6 (4%)
	Grounded Theory	154	39 (25%)	155	89 (57%)	52 (34%)	14 (9%)
	Discourse analysis	154	39 (25%)	154	93 (60%)	57 (37%)	4 (3%)
Further methods of health services research	Creation of meta-analyses	147	82 (56%)	147	29 (20%)	97 (66%)	21 (14%)
	Creation of reviews (narrative, scoping etc.)	147	61 (42%)	145	36 (25%)	67 (46%)	42 (29%)
	Creation of systematic reviews	147	53 (36%)	145	39 (27%)	71 (49%)	35 (24%)
	Complex interventions	147	53 (36%)	142	69 (49%)	55 (39%)	18 (13%)
	Patient Reported Outcomes Measures (PROM)	147	49 (33%)	147	82 (56%)	42 (29%)	23 (16%)
	Patient Reported Outcomes Experience Measures	147	41 (28%)	143	92 (64%)	42 (29%)	9 (6%)
	Carrying out real laboratories	147	37 (25%)	146	98 (67%)	41 (28%)	7 (5%)

#Multiple answers possible

Table S2: Proportion of participants with a desire for continuing education in scientific research methods and the level of knowledge and utilization of non-scientific employees

	Methods	N	Desire for continuing education#	N	No knowledge	Knowledge, but no utilization yet	Already used
Quantitative survey methods	Questionnaire development	56	32 (57%)	74	6 (8%)	25 (34%)	43 (58%)
	Questionnaire validation	56	26 (46%)	72	11 (15%)	41 (57%)	20 (28%)
	Collection of primary data	56	24 (43%)	73	12 (16%)	34 (47%)	27 (37%)
	Dealing with secondary data	56	15 (27%)	74	14 (19%)	36 (49%)	24 (32%)
Quantitative evaluation methods	Verification of model quality	56	11 (20%)	66	49 (74%)	14 (21%)	3 (5%)
	Methods of variable selection	56	10 (18%)	65	50 (77%)	13 (20%)	2 (3%)
	Structuring methods	56	9 (16%)	66	37 (56%)	22 (33%)	7 (11%)
	Correlation analyses	56	9 (16%)	66	48 (73%)	8 (12%)	10 (15%)
	Hypothesis tests	56	9 (16%)	66	39 (59%)	14 (21%)	13 (20%)
	Hierarchical models/multi-level models	56	8 (14%)	65	44 (68%)	14 (22%)	7 (11%)
	Regression analyses	56	6 (11%)	66	40 (61%)	15 (23%)	11 (17%)
Qualitative survey methods	Interviews	48	24 (50%)	51	7 (13%)	27 (53%)	17 (33%)
	Participatory observation	48	20 (42%)	51	16 (31%)	24 (47%)	11 (22%)
	Document analysis	48	19 (37%)	51	17 (33%)	21 (41%)	13 (26%)
	Group discussion method	48	15 (31%)	50	12 (24%)	30 (60%)	8 (16%)
	Participatory survey methods	48	10 (21%)	51	25 (49%)	21 (41%)	5 (10%)
Qualitative evaluation methods	Content analysis	48	14 (29%)	45	15 (33%)	19 (42%)	11 (24%)
	Discourse analysis	48	13 (27%)	46	30 (65%)	15 (33%)	1 (2%)
	Biographical research methods	48	9 (19%)	47	25 (53%)	20 (43%)	2 (4%)
	Documentary method	48	8 (17%)	47	19 (40%)	21 (45%)	7 (15%)
	Thematic analysis	48	7 (15%)	47	21 (45%)	18 (38%)	8 (17%)
	Grounded Theory	48	7 (15%)	47	32 (68%)	14 (30%)	1 (2%)
Further methods of health services research	Creation of meta-analyses	45	16 (36%)	44	20 (46%)	22 (50%)	2 (5%)
	Complex interventions	45	13 (29%)	41	25 (61%)	15 (37%)	1 (2%)
	Creation of systematic reviews	45	12 (27%)	43	21 (49%)	17 (40%)	5 (12%)
	Patient Reported Outcomes Measures (PROM)	45	12 (27%)	44	32 (73%)	10 (23%)	2 (5%)
	Creation of reviews (narrative, scoping etc.)	45	9 (20%)	43	18 (42%)	22 (51%)	3 (7%)
	Patient Reported Outcomes Experience Measures	45	4 (9%)	41	32 (78%)	9 (22%)	0 (0%)
	Carrying out real laboratories	45	3 (7%)	44	34 (77%)	10 (23%)	0 (0%)

#Multiple answers possible

Table S3: Proportion of participants with a desire for continuing education in scientific research methods as well as knowledge and utilization status not yet, but future scientific employees

	Methods	N	Desire for continuing education#	N	No knowledge	Knowledge, but no utilization yet	Already used
Quantitative survey methods	Questionnaire validation	25	13 (52%)	30	3 (10%)	15 (50%)	12 (40%)
	Questionnaire development	25	12 (48%)	30	0 (0%)	12 (40%)	18 (60%)
	Collection of primary data	25	12 (48%)	30	1 (3%)	10 (33%)	19 (63%)
	Dealing with secondary data	25	10 (40%)	30	1 (3%)	12 (40%)	17 (57%)
Quantitative evaluation methods	Correlation analyses	25	17 (68%)	28	2 (7%)	19 (68%)	7 (25%)
	Verification of model quality	25	15 (60%)	28	17 (61%)	10 (36%)	1 (4%)
	Hypothesis tests	25	14 (56%)	27	6 (22%)	10 (37%)	11 (41%)
	Regression analyses	25	14 (56%)	28	4 (14%)	17 (61%)	7 (25%)
	Methods of variable selection	25	11 (44%)	28	15 (54%)	11 (39%)	2 (7%)
	Structuring methods	25	10 (40%)	28	11 (39%)	13 (46%)	4 (14%)
	Hierarchical models/multi-level models	25	10 (40%)	28	12 (43%)	15 (54%)	1 (4%)
Qualitative survey methods	Interviews	23	12 (52%)	24	2 (8%)	9 (38%)	13 (54%)
	Group discussion method	23	12 (52%)	24	3 (13%)	14 (59%)	7 (29%)
	Document analysis	23	12 (52%)	24	2 (8%)	11 (46%)	11 (46%)
	Participatory observation	23	7 (30%)	24	8 (33%)	10 (42%)	6 (25%)
	Participatory survey methods	23	9 (39%)	24	7 (29%)	11 (46%)	6 (25%)
Qualitative evaluation methods	Content analysis	23	16 (70%)	23	7 (30%)	6 (26%)	10 (43,5%)
	Documentary method	23	10 (44%)	23	14 (61%)	6 (26%)	3 (13%)
	Thematic analysis	23	9 (39%)	23	10 (44%)	9 (39%)	4 (17%)
	Biographical research methods	23	9 (39%)	24	14 (58%)	9 (38%)	1 (4%)
	Grounded Theory	23	9 (39%)	24	14 (52%)	8 (33%)	2 (8%)
	Discourse analysis	23	9 (39%)	24	15 (63%)	8 (33%)	1 (4%)
Further methods of health services research	Creation of reviews (narrative, scoping etc.)	22	13 (59%)	22	4 (18%)	12 (55%)	6 (27%)
	Creation of meta-analyses	22	12 (55%)	22	3 (14%)	16 (73%)	3 (14%)
	Creation of systematic reviews	22	12 (55%)	22	6 (27%)	12 (55%)	4 (18%)
	Patient Reported Outcomes Measures (PROM)	22	12 (55%)	22	12 (55%)	5 (23%)	5 (23%)
	Complex interventions	22	10 (46%)	21	9 (43%)	10 (48%)	2 (10%)
	Patient Reported Outcomes Experience Measures	22	9 (41%)	22	12 (55%)	8 (36%)	2 (9%)
	Carrying out real laboratories	22	7 (32%)	21	17 (81%)	3 (14%)	1 (5%)

#Multiple answers possible

Table S4: Proportion of participants with a desire for continuing education in scientific research methods and knowledge and utilization of scientific methods for up to 3 years

	Methods	N	Desire for continuing education#	N	No knowledge	Knowledge, but no utilization yet	Already used
Quantitative survey methods	Questionnaire validation	30	17 (57%)	30	1 (3%)	20 (67%)	9 (30%)
	Dealing with secondary data	30	15 (50%)	29	2 (7%)	15 (52%)	12 (41%)
	Questionnaire development	30	13 (43%)	30	0 (0%)	13 (43%)	17 (57%)
	Collection of primary data	30	11 (37%)	29	1 (3%)	5 (17%)	23 (79%)
Quantitative evaluation methods	Hypothesis tests	30	18 (60%)	29	3 (10%)	7 (24%)	19 (66%)
	Regression analyses	30	17 (57%)	29	1 (3%)	18 (62%)	10 (35%)
	Correlation analyses	30	17 (57%)	29	2 (7%)	13 (45%)	14 (48%)
	Structuring methods	30	15 (50%)	29	4 (14%)	19 (66%)	6 (21%)
	Methods of variable selection	30	15 (50%)	28	19 (68%)	9 (32%)	0 (0%)
	Hierarchical models/multi-level models	30	14 (47%)	29	16 (55%)	11 (38%)	2 (7%)
	Verification of model quality	30	13 (43%)	29	16 (55%)	13 (45%)	0 (0%)
Qualitative survey methods	Document analysis	29	12 (41%)	29	4 (14%)	14 (48%)	11 (38%)
	Interviews	29	10 (35%)	29	0 (0%)	16 (55%)	13 (45%)
	Group discussion method	29	10 (35%)	29	1 (3%)	22 (76%)	6 (21%)
	Participatory observation	29	9 (31%)	29	4 (14%)	17 (59%)	8 (28%)
	Participatory survey methods	29	8 (28%)	29	4 (14%)	20 (69%)	5 (17%)
Qualitative evaluation methods	Grounded Theory	29	16 (55%)	29	12 (41%)	14 (48%)	3 (10%)
	Content analysis	29	12 (41%)	27	4 (15%)	9 (33%)	14 (52%)
	Discourse analysis	29	12 (41%)	29	15 (52%)	14 (48%)	0 (0%)
	Thematic analysis	29	8 (28%)	29	10 (35%)	11 (38%)	8 (28%)
	Biographical research methods	29	8 (28%)	29	11 (38%)	18 (62%)	0 (0%)
	Documentary method	29	7 (24%)	29	10 (35%)	17 (59%)	2 (7%)
Further methods of health services research	Creation of meta-analyses	28	18 (64%)	28	2 (7%)	24 (86%)	2 (7%)
	Creation of reviews (narrative, scoping etc.)	28	16 (57%)	27	3 (11%)	16 (59%)	8 (30%)
	Creation of systematic reviews	28	15 (54%)	28	4 (14%)	16 (57%)	8 (29%)
	Patient Reported Outcomes Measures (PROM)	28	13 (46%)	28	13 (46%)	13 (46%)	2 (7%)
	Complex interventions	28	11 (39%)	28	9 (32%)	15 (54%)	4 (14%)
	Patient Reported Outcomes Experience Measures	28	11 (39%)	28	16 (57%)	11 (39%)	1 (4%)
	Carrying out real laboratories	28	10 (36%)	28	11 (39%)	15 (54%)	2 (7%)

#Multiple answers possible

Table S5: Proportion of participants with a desire for continuing education in scientific research methods and knowledge and utilization level of 4 to 10 years of scientific activity

	Methods	N	Desire for continuing education#	N	No knowledge	Knowledge, but no utilization yet	Already used
Quantitative survey methods	Questionnaire validation	29	12 (41%)	32	1 (3%)	23 (72%)	8 (25%)
	Dealing with secondary data	29	12 (41%)	32	1 (3%)	10 (31%)	21 (66%)
	Questionnaire development	29	10 (35%)	31	2 (7%)	13 (42%)	16 (52%)
	Collection of primary data	29	5 (17%)	32	0 (0%)	3 (9%)	29 (91%)
Quantitative evaluation methods	Hierarchical models/multi-level models	29	21 (72%)	31	9 (29%)	14 (45%)	8 (26%)
	Structuring methods	29	20 (69%)	31	5 (16%)	13 (42%)	13 (42%)
	Verification of model quality	29	18 (62%)	31	11 (36%)	12 (39%)	8 (26%)
	Methods of variable selection	29	17 (59%)	30	15 (50%)	10 (33%)	5 (17%)
	Regression analyses	29	15 (52%)	31	3 (10%)	7 (23%)	21 (68%)
	Hypothesis tests	29	10 (35%)	31	3 (10%)	3 (10%)	25 (81%)
	Correlation analyses	29	11 (38%)	31	3 (10%)	4 (13%)	24 (77%)
Qualitative survey methods	Interviews	26	11 (42%)	28	0 (0%)	16 (57%)	12 (43%)
	Group discussion method	26	11 (42%)	27	4 (15%)	15 (56%)	8 (30%)
	Participatory observation	26	11 (42%)	28	6 (21%)	20 (71%)	2 (7%)
	Participatory survey methods	26	11 (42%)	28	5 (18%)	17 (61%)	6 (21%)
	Document analysis	26	10 (39%)	28	4 (14%)	14 (50%)	10 (36%)
Qualitative evaluation methods	Content analysis	26	11 (42%)	27	6 (22%)	11 (41%)	10 (37%)
	Thematic analysis	26	10 (39%)	27	13 (48%)	10 (37%)	4 (15%)
	Documentary method	26	10 (39%)	27	15 (56%)	9 (33%)	3 (11%)
	Grounded Theory	26	10 (39%)	27	14 (52%)	10 (37%)	3 (11%)
	Biographical research methods	26	9 (35%)	27	15 (56%)	12 (44%)	0 (0%)
	Discourse analysis	26	9 (35%)	27	17 (63%)	10 (37%)	0 (0%)
Further methods of health services research	Creation of meta-analyses	25	21 (84%)	26	2 (8%)	18 (69%)	6 (23%)
	Creation of systematic reviews	25	15 (60%)	26	3 (12%)	14 (54%)	9 (35%)
	Patient Reported Outcomes Experience Measures	25	14 (56%)	26	19 (73%)	5 (19%)	2 (8%)
	Patient Reported Outcomes Measures (PROM)	25	13 (52%)	26	16 (62%)	5 (19%)	5 (19%)
	Complex interventions	25	11 (44%)	26	13 (50%)	7 (27%)	6 (23%)
	Carrying out real laboratories	25	11 (44%)	26	19 (73%)	6 (23%)	1 (4%)
	Creation of reviews (narrative, scoping etc.)	25	9 (36%)	26	6 (23%)	11 (42%)	9 (35%)

#Multiple answers possible

Table S6: Proportion of participants with a desire for continuing education in scientific research methods and a level of knowledge and utilization already more than 10 years of scientific activity

	Methods	N	Desire for continuing education #	N	No knowledge	Knowledge, but no utilization yet	Already used
Quantitative survey methods	Questionnaire validation	30	5 (17%)	33	1 (3%)	25 (76%)	7 (21%)
	Questionnaire development	30	5 (17%)	33	0 (0%)	18 (55%)	15 (46%)
	Dealing with secondary data	30	5 (17%)	33	0 (0%)	12 (40%)	18 (60%)
	Collection of primary data	30	2 (7%)	33	0 (0%)	9 (27%)	24 (73%)
Quantitative evaluation methods	Verification of model quality	30	14 (47%)	32	8 (25%)	16 (50%)	8 (25%)
	Structuring methods	30	13 (43%)	32	4 (13%)	12 (38%)	16 (50%)
	Hierarchical models/multi-level models	30	13 (43%)	32	10 (31%)	17 (53%)	5 (16%)
	Methods of variable selection	30	12 (40%)	32	8 (25%)	17 (53%)	7 (22%)
	Regression analyses	30	10 (33%)	32	1 (3%)	5 (16%)	26 (81%)
	Correlation analyses	30	8 (27%)	32	1 (3%)	8 (25%)	23 (72%)
	Hypothesis tests	30	8 (27%)	31	0 (0%)	4 (13%)	27 (87%)
Qualitative survey methods	Document analysis	28	6 (21%)	30	4 (13%)	14 (47%)	12 (40%)
	Participatory survey methods	28	4 (14%)	30	10 (33%)	14 (47%)	6 (20%)
	Interviews	28	3 (11%)	30	4 (13%)	17 (57%)	9 (30%)
	Participatory observation	28	3 (11%)	30	8 (27%)	17 (57%)	5 (17%)
	Group discussion method	28	2 (7%)	29	10 (35%)	13 (45%)	6 (21%)
Qualitative evaluation methods	Discourse analysis	28	6 (21%)	28	16 (57%)	10 (36%)	2 (7%)
	Content analysis	28	6 (21%)	27	13 (48%)	7 (26%)	7 (26%)
	Thematic analysis	28	5 (18%)	28	15 (54%)	6 (21%)	7 (25%)
	Documentary method	28	5 (18%)	28	17 (61%)	6 (21%)	5 (18%)
	Biographical research methods	28	4 (14%)	28	17 (61%)	8 (29%)	3 (11%)
	Grounded Theory	28	4 (14%)	28	17 (61%)	6 (21%)	5 (18%)
Further methods of health services research	Creation of meta-analyses	27	15 (56%)	27	2 (7%)	17 (63%)	8 (30%)
	Creation of systematic reviews	27	7 (26%)	26	5 (19%)	12 (46%)	9 (35%)
	Creation of reviews (narrative, scoping etc.)	27	6 (22%)	27	5 (19%)	6 (22%)	16 (59%)
	Carrying out real laboratories	27	6 (22%)	27	17 (63%)	7 (26%)	3 (11%)
	Complex interventions	27	4 (15%)	26	13 (50%)	8 (31%)	5 (19%)
	Patient Reported Outcomes Measures (PROM)	27	3 (11%)	27	9 (33%)	9 (33%)	9 (33%)
	Patient Reported Outcomes Experience Measures	27	3 (11%)	26	13 (50%)	9 (35%)	4 (15%)

#Multiple answers possible

Table S7: The three most frequently mentioned methods used overall and grouped by participants' scientific experience

	Methods	N	Already used
Overall			
1.	Collection of primary data	197	129 (66%)
2.	Questionnaire development	198	109 (55%)
3.	Hypothesis tests	184	95 (52%)
Group: Not scientifically active (incl. "Don't know" whether scientific activity is planned)			
1.	Questionnaire development	74	43 (58%)
2.	Collection of primary data	73	27 (37%)
3.	Interviews	51	17 (33%)
Group: Not yet, but scientifically active in the future			
1.	Collection of primary data	30	19 (63%)
2.	Questionnaire development	30	18 (60%)
3.	Dealing with secondary data	30	17 (57%)
Group: Already scientifically active for up to 3 years			
1.	Collection of primary data	29	23 (79%)
2.	Hypothesis tests	29	19 (66%)
3.	Questionnaire development	30	17 (57%)
Group: Already scientifically active for 4 to 10 years			
1.	Collection of primary data	32	29 (91%)
2.	Hypothesis tests	31	25 (81%)
3.	Correlation analyses	31	24 (77%)
Group: Already scientifically active for over 10 years			
1.	Hypothesis tests	32	26 (81%)
2.	Regression analyses	32	26 (81%)
3.	Collection of primary data	33	24 (73%)

Table S8: Requests for continuing education with regard to research-related topics overall (N=136)

	Methods	Desire for continuing education#
Other research-related topics	Project coordination	78 (57%)
	Third-party funding application/ administration/ overview	63 (46%)
	Science communication	61 (45%)
	Scientific writing	55 (40%)
	Publishing in scientific journals	51 (38%)
	DFG-Application	48 (35%)
	Development of research questions	48 (35%)
	Evaluation methods	48 (35%)
	Research ethics and legal issues	46 (34%)
	EU-Application	39 (29%)
	Epidemiology	38 (28%)
	Data protection issues in research	38 (28%)
	BMBF-Application	36 (27%)
	Choice of study design	35 (26%)
	Evaluation of complex interventions	35 (26%)
	Creation of posters	33 (24%)
	Health economy	33 (24%)
	Process evaluation - models and practical approaches	31 (23%)
	Topics related to health services research (settings, methods, relevance)	26 (19%)

#Multiple answers possible

Table S9: Requests for continuing education on research-related topics for non-scientific employees (N=38)

	Methods	Desire for continuing education#
Research-related topics	Project coordination	18 (47%)
	Science communication	15 (40%)
	Scientific writing	15 (40%)
	Third-party funding application/ administration/ overview	14 (37%)
	Health economy	13 (34%)
	Evaluation methods	12 (32%)
	Creation of posters	10 (26%)
	Epidemiology	9 (24%)
	Research ethics and legal issues	9 (24%)
	Development of research questions	7 (18%)
	Data protection issues in research	6 (16%)
	DFG-Application	6 (16%)
	Publishing in scientific journals	6 (16%)
	Choice of study design	6 (16%)
	Topics related to health services research (settings, methods, relevance)	5 (13%)
	BMBF-Application	4 (11%)
	Evaluation of complex interventions	4 (11%)
	Process evaluation - models and practical approaches	4 (11%)
	EU-Application	3 (8%)

#Multiple answers possible

Table S10: Requests for continuing education on research-related topics for not yet, but future scientific employees (N=20)

	Methods	Desire for continuing education#
Research-related topics	Project coordination	12 (60%)
	Development of research questions	11 (55%)
	Scientific writing	11 (55%)
	Publishing in scientific journals	10 (50%)
	Choice of study design	10 (50%)
	DFG-Application	9 (45%)
	Third-party funding application/ administration/ overview	9 (45%)
	Evaluation methods	9 (45%)
	EU-Application	8 (40%)
	Evaluation of complex interventions	8 (40%)
	Research ethics and legal issues	8 (40%)
	Process evaluation - models and practical approaches	8 (40%)
	Topics related to health services research (settings, methods, relevance)	7 (35%)
	Science communication	7 (35%)
	BMBF-Application	6 (30%)
	Data protection issues in research	6 (30%)
	Epidemiology	6 (30%)
	Creation of posters	6 (30%)
	Health economy	5 (25%)

#Multiple answers possible

Table S11: Requests for continuing education on research-related topics for employees with up to 3 years of scientific activity (N=27)

	Methods	Desire for continuing education#
Research-related topics	Development of research questions	18 (67%)
	Project coordination	18 (67%)
	Publishing in scientific journals	16 (59%)
	Third-party funding application/ administration/ overview	15 (56%)
	Evaluation methods	15 (56%)
	Data protection issues in research	13 (48%)
	Scientific writing	13 (48%)
	Science communication	13 (48%)
	Research ethics and legal issues	11 (41%)
	DFG-Application	10 (37%)
	Creation of posters	10 (37%)
	Evaluation of complex interventions	10 (37%)
	Process evaluation - models and practical approaches	9 (33%)
	BMBF-Application	8 (30%)
	Choice of study design	8 (30%)
	Epidemiology	6 (22%)
	Health economy	6 (22%)
	EU-Application	5 (19%)
	Topics related to health services research (settings, methods, relevance)	4 (15%)

#Multiple answers possible

Table S12: Requests for continuing education on research-related topics for employees with 4 to 10 years of scientific activity (N=25)

	Methods	Desire for continuing education#
Research-related topics	Third-party funding application/ administration/ overview	16 (64%)
	Data protection issues in research	14 (56%)
	DFG-Application	14 (56%)
	Project coordination	14 (56%)
	Science communication	14 (56%)
	Publishing in scientific journals	13 (52%)
	EU-Application	12 (48%)
	Scientific writing	12 (48%)
	BMBF-Application	9 (36%)
	Evaluation of complex interventions	9 (36%)
	Research ethics and legal issues	9 (36%)
	Epidemiology	7 (28%)
	Evaluation methods	7 (28%)
	Development of research questions	7 (28%)
	Process evaluation - models and practical approaches	7 (28%)
	Choice of study design	7 (28%)
	Topics related to health services research (settings, methods, relevance)	6 (24%)
	Creation of posters	4 (16%)
	Health economy	4 (16%)

#Multiple answers possible

Table S13: Requests for continuing education on research-related topics for employees with over 10 years of scientific activity (N=26)

	Methods	Desire for continuing education#
Research-related topics	Project coordination	16 (62%)
	Data protection issues in research	12 (46%)
	Science communication	12 (46%)
	EU-Application	11 (42%)
	Epidemiology	10 (39%)
	BMBF-Application	9 (35%)
	DFG-Application	9 (35%)
	Third-party funding application/ administration/ overview	9 (35%)
	Research ethics and legal issues	9 (35%)
	Publishing in scientific journals	6 (23%)
	Evaluation methods	5 (19%)
	Development of research questions	5 (19%)
	Health economy	5 (19%)
	Evaluation of complex interventions	4 (15%)
	Topics related to health services research (settings, methods, relevance)	4 (15%)
	Choice of study design	4 (15%)
	Scientific writing	4 (15%)
	Creation of posters	3 (12%)
	Process evaluation - models and practical approaches	3 (12%)

#Multiple answers possible

Table S14: Requests for continuing education regarding data processing software overall

	Methods	N	Desire for continuing education#
Statistical analysis programs	SPSS	130	82 (63%)
	SAS	115	48 (42%)
	STATA	118	41 (37%)
Qualitative evaluation programs	MAXQDA	110	44 (37%)
	Atlas.ti	112	26 (23%)
	F4	109	23 (21%)

#Multiple answers possible

Table S15: Requests for continuing education regarding data processing software for non-scientific employees

	Methods	N	Desire for continuing education#
Statistical analysis programs	SPSS	38	18 (47%)
	SAS	35	11 (31%)
	STATA	35	13 (37%)
Qualitative evaluation programs	MAXQDA	35	10 (29%)
	Atlas.ti	34	7 (21%)
	F4	33	8 (24%)

#Multiple answers possible

Table S16: Requests for continuing education regarding data processing software for not yet, but future scientific employees

	Methods	N	Desire for continuing education#
Statistical analysis programs	SPSS	20	14 (70%)
	SAS	17	7 (41%)
	STATA	14	7 (50%)
Qualitative evaluation programs	MAXQDA	16	7 (44%)
	Atlas.ti	15	5 (33%)
	F4	15	6 (40%)

#Multiple answers possible

Table S17: Requests for continuing education regarding data processing software for employees with up to 3 years of scientific activity

	Methods	N	Desire for continuing education#
Statistical analysis programs	SPSS	25	19 (76%)
	SAS	21	8 (38%)
	STATA	21	7 (33%)
Qualitative evaluation programs	MAXQDA	24	13 (54%)
	Atlas.ti	21	5 (24%)
	F4	21	5 (24%)

#Multiple answers possible

Table S18: Requests for continuing education regarding data processing software for employees with 4 to 10 years of scientific activity

	Methods	N	Desire for continuing education#
Statistical analysis programs	SPSS	24	18 (75%)
	SAS	22	12 (55%)
	STATA	20	6 (30%)
Qualitative evaluation programs	MAXQDA	23	9 (39%)
	Atlas.ti	22	6 (27%)
	F4	21	2 (10%)

#Multiple answers possible

Table S19: Requests for continuing education regarding data processing software for employees with over 10 years of scientific activity

	Methods	N	Desire for continuing education#
Statistical analysis programs	SPSS	23	13 (57%)
	SAS	20	10 (50%)
	STATA	20	6 (30%)
Qualitative evaluation programs	MAXQDA	20	5 (25%)
	Atlas.ti	20	3 (15%)
	F4	19	2 (11%)

#Multiple answers possible

Table S20: The three most frequently mentioned continuing education requests regarding research software overall and grouped according to the participants' scientific experience

	Methods	N	Desire for continuing education
Overall			
1.	SPSS	130	82 (63%)
2.	SAS	115	48 (42%)
3.	STATA<br>MAXQDA	118<br>110	41 (37%)<br>44 (37%)
Group: Not scientifically active (incl. "Don't know" whether scientific activity is planned)			
1.	SPSS	38	18 (47%)
2.	STATA	35	13 (37%)
3.	SAS	35	11 (31%)
Group: Not yet, but scientifically active in the future			
1.	SPSS	20	14 (70%)
2.	STATA	14	7 (50%)
3.	MAXQDA	16	7 (44%)
Group: Already scientifically active for up to 3 years			
1.	SPSS	25	19 (76%)
2.	MAXQDA	24	13 (54%)
3.	SAS	21	8 (38%)
Group: Already scientifically active for 4 to 10 years			
1.	SPSS	24	18 (75%)
2.	SAS	22	12 (55%)
3.	MAXQDA	23	9 (39%)
Group: Already scientifically active for over 10 years			
1.	SPSS	23	13 (57%)
2.	SAS	20	10 (50%)
3.	STATA	20	6 (30%)

Table S21: Desired framework conditions of the continuing education courses overall and grouped according to the scientific experience of the participants

	Methods	N	n (%)
Overall			
Desired organization of the training	Blended learning programs	136	57 (42%)
	Online		32 (24%)
	Face-to-face		27 (20%)
	No preference		20 (15%)
Request for certificate of participation	Yes	136	116 (85%)
	No		1 (1%)
	No preference		19 (14%)
Group: Not scientifically active (incl. "Don't know" whether scientific activity is planned)			
Desired organization of the training	Face-to-face	38	14 (37%)
	Blended learning programs		11 (29%)
	Online		5 (13%)
	No preference		8 (21%)
Request for certificate of participation	Yes	38	31 (82%)
	No		0 (0%)
	No preference		7 (18%)
Group: Not yet, but scientifically active in the future			
Desired organization of the training	Blended learning programs	20	10 (50%)
	Online		5 (25%)
	Face-to-face		3 (15%)
	No preference		2 (10%)
Request for certificate of participation	Yes	20	16 (80%)
	No		1 (5%)
	No preference		3 (15%)
Group: Already scientifically active for up to 3 years			
Desired organization of the training	Online	27	11 (41%)
	Blended learning programs		10 (37%)
	Face-to-face		3 (11%)
	No preference		3 (11%)
Request for certificate of participation	Yes	27	24 (89%)
	No		0 (0%)
	No preference		3 (11%)
Group: Already scientifically active for 4 to 10 years			
Desired organization of the training	Blended learning programs	25	14 (56%)
	Online		5 (20%)
	Face-to-face		3 (12%)
	No preference		3 (12%)
Request for certificate of participation	Yes	25	22 (88%)
	No		0 (0%)
	No preference		3 (12%)
Group: Already scientifically active for over 10 years			
Desired organization of the training	Blended learning programs	26	12 (46%)
	Online		6 (23%)
	Face-to-face		4 (15%)
	No preference		4 (15%)
Request for certificate of participation	Yes	26	23 (89%)
	No		0 (0%)
	No preference		3 (12%)
